# Ninjin’yoeito for Impaired Oral Function in Older Adults: A Prospective, Open-Label Pilot Study

**DOI:** 10.3390/medicina62010048

**Published:** 2025-12-26

**Authors:** Quang Trung Ngo, Akiko Shirai, Hongyang Li, Akiyoshi Takami, Akihiro Kawahara, Lian Liang, Tomokazu Yoshizaki, Keiko Ogawa-Ochiai

**Affiliations:** 1 Kampo Clinical Center, Hiroshima University Hospital, 1 Chome-2-3 Kasumi, Minami Ward, Hiroshima 734-8551, Japan; nqtrung@hpmu.edu.vn (Q.T.N.); hoyalee328@yahoo.co.jp (H.L.); abcd@hiroshima-u.ac.jp (A.K.); lianglian96@gmail.com (L.L.); 2 Faculty of Traditional Medicine, Hai Phong University of Medicine and Pharmacy, Hai Phong City 180000, Vietnam; 3 Division of Otolaryngology-Head and Neck Surgery, Graduate School of Medical Science, Kanazawa University, Kanazawa 920-8640, Japan; acorin.to4987@gmail.com (A.S.); tomoy@med.kanazawa-u.ac.jp (T.Y.); 4 Department of Internal Medicine, Division of Hematology, School of Medicine, Aichi Medical University, Karimata-1-1 Yazako, Nagakute 480-1195, Japan; takami-knz@umin.ac.jp

**Keywords:** Ninjin’yoeito, Kampo medicine, oral health, oral frailty, elderly, frailty, pilot study, traditional Japanese medicine, swallowing, xerostomia

## Abstract

*Background and Objectives:* Japan’s aging population faces growing challenges related to oral frailty, a condition characterized by the decline of oral function associated with physical and nutritional deterioration. Impaired oral function contributes to reduced chewing, swallowing, and saliva secretion, leading to poor appetite and frailty progression. Ninjin’yoeito (NYT), a traditional Kampo formula, has been clinically used to improve systemic weakness and oral symptoms. This study aimed to evaluate the efficacy and safety of NYT in improving oral health among elderly individuals with impaired oral function. *Materials and Methods:* In this open-label prospective study, patients received NYT daily for 12 weeks. Assessments included oral symptom scores, mucosal moisture, repetitive saliva swallowing tests (RSST), gustatory function by visual analogue scale (VAS), an 11-item oral questionnaire, and immune profiling by flow cytometry. Safety was assessed through hematological and biochemical tests. *Results*: Symptom scores decreased from 8.27 at baseline to 3.64 at 12 weeks (*p* = 0.006), while oral condition scores improved from 5.09 to 1.36 (*p* = 0.006). Mucosal moisture increased (25.1 to 28.1, *p* = 0.03), and RSST frequency improved (2.18 to 4.55, *p* = 0.046). Questionnaire scores declined from 5.1 to 2.0 (*p* < 0.001). VAS-taste was unchanged overall (*p* = 0.21) but improved in low baseline patients. Laboratory findings showed no adverse changes, with favorable lipid trends. Immune analysis revealed a decline in NKG2D expression (*p* = 0.02), whereas other activating and inhibitory markers remained stable. *Conclusions*: NYT was well tolerated and associated with gradual improvements in oral and physical symptoms among elderly individuals with impaired oral function. These findings provide preliminary evidence supporting the feasibility of Kampo-based approaches for maintaining oral health in aging populations and warrant further validation in larger controlled trials.

## 1. Introduction

Japan is currently experiencing a rapid demographic shift toward a super-aged society, in which maintaining oral function has become an essential component of elderly healthcare [1]. Oral frailty—defined as a decrease in oral function accompanied by a decrease in mental and physical function —has recently emerged as a critical public health issue [2,3]. This condition not only impairs chewing, swallowing, and saliva secretion but also contributes to nutritional decline, weight loss, and frailty progression among older adults [4,5,6,7,8]. Previous epidemiological studies have reported that more than 20–30% of Japanese individuals aged ≥ 75 years experience some degree of oral frailty, highlighting its high prevalence and impact on quality of life [9].

Oral function is closely related to overall health, nutritional status, and longevity. Decreased salivary flow, dry mouth, and swallowing difficulty can reduce food intake and digestion efficiency, leading to malnutrition and increased mortality risk [7,8,10]. Furthermore, poor oral function affects communication, social interaction, and psychological well-being, which are key components of healthy aging [3,11,12]. Aging is also accompanied by immunosenescence and metabolic decline, which increase vulnerability to diseases and further exacerbate frailty [13,14]. Therefore, maintaining oral function and nutritional balance is considered a cornerstone of frailty prevention and comprehensive geriatric care.

Ninjin’yoeito (NYT), also known as Ren Shen Yang Rong Tang in Traditional Chinese Medicine, is a classical Kampo prescription approved by the Japanese Ministry of Health, Labour and Welfare. NYT consists of extracts from 12 medicinal herbs: *Ginseng radix*, *Atractylodis macrocephalae rhizoma*, *Poria*, *Glycyrrhizae radix*, *Angelicae radix*, *Rehmanniae radix*, *Astragali radix*, *Cinnamomi cortex*, *Paeoniae radix*, *Citrus unshiu* peel, *Schisandrae fructus*, and *Polygalae radix*. NYT has been prescribed since the 16th century in Japan and other Asian countries for a wide range of conditions, including weakness and neuropathic pain [15], as well as asthenia and general debility [16]. It has also been reported to improve the nutritional status of frail older adults [17]. Regarding oral function, Miyazaki et al. demonstrated that NYT alleviated oxybutynin hydrochloride-induced xerostomia in patients with psychogenic urinary frequency or unstable bladder [18], and additional case reports have described improvements in dry mouth and appetite following two months of treatment [19]. Moreover, experimental studies have shown that NYT may modulate immune responses, including effects on regulatory T-cell populations [20]. These findings lead us to believe that NYT may help enhance physical strength, oral health, nutritional status, and immune balance in elderly individuals, supporting its potential as a multifaceted therapeutic option.

However, evidence regarding its impact on oral function and nutrition in older adults remains limited, and few studies have comprehensively examined its safety profile through hematological and biochemical parameters. Therefore, the present study was designed as an exploratory, open-label pilot trial to evaluate the potential of NYT in improving oral health among elderly individuals. In addition to assessing preliminary efficacy and safety, this study also aimed to examine the feasibility of long-term follow-up in this population, providing groundwork for future large-scale clinical studies focusing on geriatric oral health promotion.

## 2. Materials and Methods

### 2.1. Participants

This exploratory, open-label pilot study evaluated the feasibility and preliminary effects of NYT in older adults with impaired oral function. Participants aged ≥ 65 years were recruited at Kanazawa University Hospital between July 2019 and September 2022 using convenience sampling, which is appropriate for pilot feasibility research. In accordance with the CONSORT 2010 Extension for Pilot and Feasibility Trials, a formal sample size calculation was not performed, as the primary objective was to assess feasibility rather than to test predefined hypotheses. Impaired oral function was defined according to standardized Japanese oral frailty criteria. Participants were required to meet at least one of the following conditions: (1) two or more positive responses on a three-item screening questionnaire (“difficulty chewing hard foods,” “coughing or choking when drinking,” and “oral dryness”); (2) abnormal clinical oral findings, including poor oral hygiene, oral malodor, reduced facial or oral expressiveness, articulation difficulties, or residual food after swallowing; or (3) a RSST score < 3. Suitability for NYT administration was further determined based on Kampo diagnostic principles [21]. Participants were considered appropriate if they exhibited classical qi-and-blood deficiency patterns, such as post-illness fatigue, reduced vitality, appetite loss, cold extremities, night sweats, or anemia [22,23]. Oral status was clinically evaluated to confirm that all participants had adequate natural dentition or prosthetic support to perform chewing, swallowing, and salivary assessments, although detailed prosthodontic classifications were not systematically recorded.

Eligibility assessments were independently conducted by two experienced physicians using the Severity of Symptoms Questionnaire and the Oral Condition Questionnaire, with discrepancies resolved by consensus. Exclusion criteria included hypersensitivity to NYT, severe acute illness, significant hepatic or renal dysfunction, active cancer treatment, use of conflicting medications, and inability to comply with study procedures. All participants provided written informed consent. The study adhered to the Declaration of Helsinki and was approved by the Medical Ethics Committee of Kanazawa University (approval no. 000039882; approval date: 28 March 2019; UMIN ID: UMIN000039882).

A total of 11 participants aged > 65 years (9 females and 2 males; mean age, 76.4 ± 5.7 years; range, 70–88 years) were enrolled. The mean height was 156.2 ± 8.6 cm (range, 143–173 cm), and participants had an average of 3.5 comorbidities. The most common comorbid conditions were hypertension (36.4%), dyslipidemia (36.4%), type 2 diabetes mellitus (18.2%), glaucoma (18.2%), allergic rhinitis (18.2%), and insomnia (18.2%). Baseline demographic and clinical characteristics of the participants are summarized in Table 1.

### 2.2. Clinical and Oral Assessment Methods

Clinical and oral conditions were assessed using a combination of objective measurements and patient-reported questionnaires. Symptom severity was evaluated using the Severity of Symptoms Questionnaire, while oral conditions were assessed using the Oral Condition Questionnaire. Oral mucosal moisture was objectively measured using a MOUKAS oral hygrometer (Life Co., Ltd., Saitama, Japan). Swallowing function was assessed using the RSST. The complete Severity of Symptoms Questionnaire and Oral Condition Questionnaire are provided in Supplementary Tables S1 and S2.

The Severity of Symptoms Questionnaire and the Oral Condition Questionnaire were developed by the clinical team based on Japanese oral frailty guidelines. Although these instruments have not undergone formal psychometric validation, conceptually similar validated tools have been reported in previous studies, including the 8-item Oral Frailty Index developed by Nomura et al. [24], and 11-item oral frailty screening instruments evaluated in geriatric cohorts [25]. These referenced instruments are cited for contextual comparison only and were not directly used in the present study. Therefore, assessments based on the questionnaires developed for this study are interpreted as exploratory.

Additional patient-reported assessments included gustatory function, evaluated using VAS, and an 11-item oral function questionnaire. This 11-item oral function questionnaire is a self-reported instrument distinct from the 11-item oral frailty screening tools described in previous studies [22]. It is widely used in clinical practice in Japan but has not been formally validated for oral frailty assessment, and this limitation is acknowledged. The full questionnaire is provided as Supplementary Table S3. The latter is widely used in clinical practice in Japan; however, its lack of formal validation for oral frailty assessment is acknowledged.

Immunological assessments were conducted on an exploratory basis using flow cytometry, including analysis of lymphocyte subsets and the expression of activating and inhibitory receptors, such as NKG2D, NKp46, PD-1, and TIM-3.

### 2.3. Evaluation of NYT Treatment on Symptom Severity

The severity of six symptoms (post-illness fatigue, irritability, appetite loss, somnolence, cold extremities, and anemia) was assessed at baseline and weeks 4, 8, and 12 using the Six-Symptom Severity Questionnaire developed by the study clinicians. Symptoms were scored as 3 (severe), 2 (moderate), 1 (mild), or 0 (none), and total scores were calculated at each time point. This questionnaire has not been psychometrically validated, nor has its reliability been established in prior studies. To improve assessment consistency, two senior physicians independently rated all items, resolving disagreements by consensus. The absence of formal validation remains a methodological limitation of this pilot study. A sample questionnaire is shown in Supplementary Table S1.

### 2.4. Evaluation of NYT Treatment Oral Function Symptoms by Physician

Oral function was assessed using an Oral Condition Questionnaire at baseline and after 4, 8, and 12 weeks of treatment. The questionnaire evaluates oral conditions and observable findings, including visible oral hygiene-related findings (such as deposits or discoloration on teeth, dentures, or the tongue), oral malodor, poor facial expressions, speech problems (e.g., unclear pronunciation, dysarthria, and reduced speech), and food residue after swallowing. According to Kampo diagnostic principles, these observable oral findings are considered clinically relevant because they may reflect impaired digestive function, reduced vital energy (qi), and decreased self-care ability, which are associated with age-related physiological characteristics [23].

Each symptom was scored by a physician as follows: 3 points for severe, 2 points for moderate, 1 point for mild, and 0 points for no signs. The total score for the five symptoms was recorded at each time point and compared across assessments. A sample of the questionnaire is provided in Supplementary Table S2.

### 2.5. Evaluation of NYT Treatment on Oral Mucosal Moisture

Oral mucosal moisture was evaluated using a portable oral hygrometer (MOUKAS Oral Hygrometer; Life Co., Ltd., Saitama, Japan). Measurements were performed at the oral mucosa under standardized conditions, with the probe gently placed in contact with the mucosal surface for a few seconds until the moisture value was automatically recorded according to the manufacturer’s instructions. Dry mouth was defined as a hygrometer value below 28.0. Assessments were conducted at baseline and after 4, 8, and 12 weeks of treatment.

### 2.6. Evaluation of NYT Treatment on Swallowing Frequency Using the Repetitive Saliva Swallowing Test

The RSST was conducted by gently placing the examiner’s second and third fingers on the patient’s laryngeal prominence and hyoid bone. The patient was instructed to swallow saliva as many times as possible within 30 s. The examiner recorded the number of times the laryngeal protuberance passed under the second finger during this period, which was noted as the RSST swallowing frequency. An RSST score of <3 swallows indicated impaired swallowing function [26]. RSST measurements were conducted at baseline and after 4, 8, and 12 weeks of NYT treatment.

### 2.7. Visual Analogue Scale (VAS) for Taste

Gustatory function was assessed using a 100 mm VAS. Participants were instructed to rate their current subjective taste perception on a straight horizontal line anchored at 0 mm (“no taste perception”) and 100 mm (“usual taste perception”), with the latter reflecting their own typical or desirable taste ability rather than an objective normative standard. Each participant marked the point on the line that best represented their perceived taste function at the time of assessment, and the distance in millimeters from the 0 mm anchor was recorded as the VAS score. Evaluations were conducted at baseline and at 4, 8, and 12 weeks of NYT treatment.

### 2.8. Patient-Reported Oral Function Questionnaire

Oral function was additionally evaluated using an 11-item patient-reported questionnaire assessing multiple domains relevant to oral frailty, including difficulties in chewing hard foods, swallowing, speaking, oral dryness, halitosis, taste recognition, and food residue. Each item was scored as 1 point for “yes” (impaired) and 0 points for “no” (normal), yielding a total score ranging from 0 (no impairment) to 11 (severe impairment). Assessments were performed at baseline and at 4, 8, and 12 weeks. A sample of the questionnaire is provided in Supplementary Table S3.

### 2.9. Hematological and Biochemical Safety Assessment

Safety was assessed using routine hematological and biochemical examinations conducted at baseline and after 12 weeks of NYT treatment. Hematological parameters included white blood cell count, red blood cell count, hemoglobin (Hb), hematocrit (Ht), platelet count, mean corpuscular volume (MCV), mean corpuscular hemoglobin (MCH), mean corpuscular hemoglobin concentration (MCHC), red cell distribution width (RDW), mean platelet volume (MPV), platelet distribution width (PDW), and differential leukocyte counts (neutrophils, eosinophils, basophils, lymphocytes, and monocytes). Biochemical parameters included C-reactive protein (CRP), aspartate aminotransferase (AST), alanine aminotransferase (ALT), γ-glutamyl transpeptidase (γ-GTP), lactate dehydrogenase (LDH), total bilirubin (T-Bil), serum electrolytes (Na, K, Cl), creatinine (CRE), blood urea nitrogen (BUN), lipid profile [low-density lipoprotein cholesterol (LDL-C), high-density lipoprotein cholesterol (HDL-C), triglycerides (TG)], and blood pressure. Changes from baseline were analyzed to monitor potential adverse effects of NYT treatment.

### 2.10. Immunological Assessment Through Blood Tests

To evaluate immune system changes, flow cytometry was used to quantify immune cells and their biomarkers before and after 12 weeks of NYT treatment. Blood samples were collected in 3.5 mL ethylenediaminetetraacetic acid tubes and centrifuged at 3000× *g* for 8 min to separate the serum. Peripheral blood mononuclear cells (PBMCs) were isolated from the remaining blood through gradient centrifugation. A portion of the PBMCs from each sample was cryopreserved for later use. PBMCs were stained with antibodies specific to cell surface markers for natural killer (NK) lymphocytes and T cells, including the following: anti-CD3, CD4, CD8, CD56, NKG2D, NKp46, NKp30, TLR4, DNAM-1, NKG2A, 4-1BB, ICOS, OX40, GITR, PD-1, CTLA4, LAG3, TIGIT, and TIM3 (BioLegend, San Diego, CA, USA). The stained cells were analyzed using a BD FACSCanto II flow cytometer (BD Biosciences, San Jose, CA, USA), and the data were analyzed using FlowJo software (version 10; Tree Star, Ashland, OR, USA).

### 2.11. Dietary NYT Supplementation

The Kampo formula NYT used in this study was Kracie Ninjinyoeito Extract Fine Granules (KB-108, Kracie Pharmaceutical Co., Tokyo, Japan), a Japanese-standard Kampo product classified under product number 875200, approval number: 16100AMZ03510000, and YJ code: 5200117C1043. Each 7.5 g daily dose (two packets) contains 3.75 g of Ninjinyoeito extract powder (Kracie Pharmaceutical Co., Ltd., 3-20-20 Kaigan, Minato-ku, Tokyo, Japan [108-8080]). The product is a dry extract mixture, light brown to brown in color, with component ratios as listed in Table 2.

### 2.12. Statistical Analysis

Given the exploratory pilot design and the small sample size, all statistical analyses were conducted with the primary objective of identifying preliminary trends rather than performing confirmatory hypothesis testing. Data obtained from the severity and oral function questionnaires, oral mucosal moisture measurements, RSST tests, VAS scores, and the 11-item oral questionnaire were first examined for normality using the Shapiro–Wilk test. Variables that were not normally distributed were analyzed using the non-parametric Friedman test with Bonferroni-adjusted post hoc pairwise comparisons. For normally distributed variables, repeated-measures ANOVA was used; however, these results were interpreted cautiously within an exploratory framework. Linear mixed-effects models (LMMs) were included only to illustrate time-dependent changes and to estimate within-subject and between-subject variability. These models were not used for confirmatory inference, thereby reducing the risk of type I and type II errors associated with high-dimensional modeling in small samples. For immune-cell subset markers and laboratory parameters (hematological and biochemical tests), paired *t*-tests or Wilcoxon signed-rank tests were applied according to data distribution. Because multiple immune markers were evaluated, the Benjamini–Hochberg false discovery rate (FDR) correction was applied. Global FDR-adjusted *p*-values are reported in Supplementary Table S4, and, for biologically related immunological pathways (T-cell subsets, activating NK receptors, inhibitory checkpoints, co-stimulatory receptors, and TLR4), subgroup-specific FDR-adjusted *p*-values are presented in Supplementary Table S4 to avoid over-correction across unrelated markers in this exploratory pilot analysis. All statistical tests were two-sided, and *p* < 0.05 was considered statistically significant within the context of exploratory analysis. Statistical analyses were performed using SPSS Statistics version 28 (IBM, Armonk, NY, USA).

Because of the small sample size and the imbalance in sex distribution (only two male participants), formal adjustment for covariates such as age and gender was not statistically feasible. Multivariable modeling was avoided to prevent unstable coefficient estimates and overfitting. Accordingly, analyses focused on within-subject comparisons without covariate adjustment, and the inability to control for age and sex effects is acknowledged as a methodological limitation of this exploratory pilot study.

## 3. Results

### 3.1. Study Design

**Figure 1:**
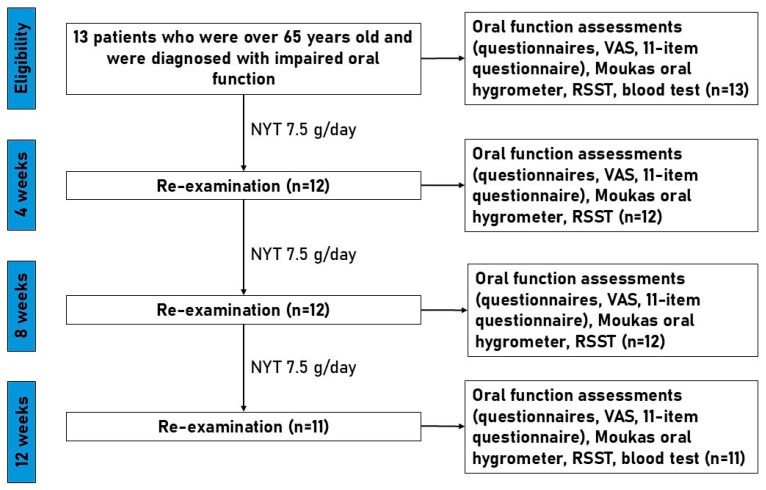
Flowchart of study design. NYT, Ninjinyoeito; RSST, repetitive saliva swallowing test.

Figure 1 illustrates the study design and timeline for patients with impaired oral function who received NYT (7.5 g/day) for 12 weeks. Eligible participants (*n* = 13) were assessed at baseline and re-examined at 4, 8, and 12 weeks. Assessments included symptom questionnaires (severity, oral function, 11-item questionnaire, and VAS), oral mucosal moisture (Moukas hygrometer), and RSST. Blood and biochemical parameters were measured at baseline and week 12. During follow-up visits, 2 patients did not attend re-examinations due to personal reasons, resulting in *n* = 12 at weeks 4 and 8, and *n* = 11 at week 12.

### 3.2. Changes in Primary Outcomes After NYT Treatment

**Figure 2:**
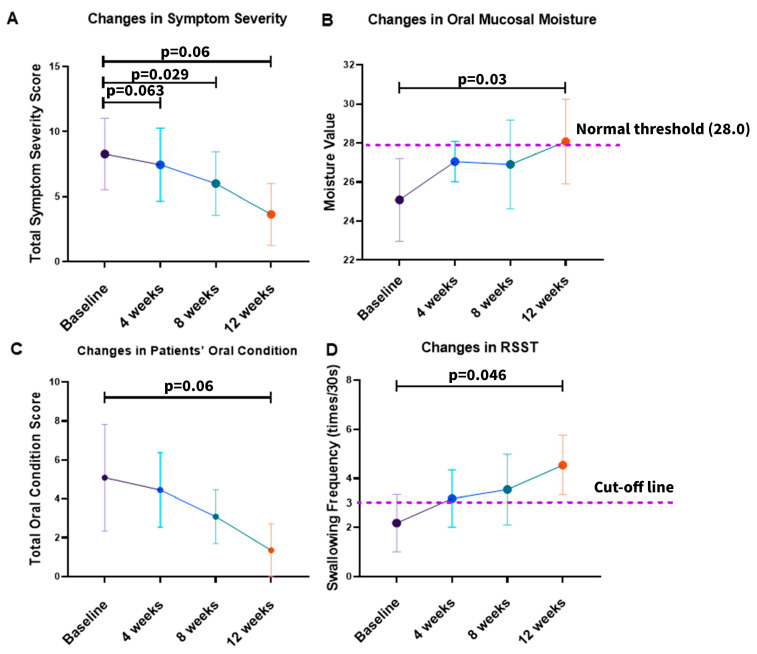
Changes in primary outcomes during NYT treatment: (**A**) total symptom severity score; (**B**) oral mucosal moisture measured by the MOUKAS Oral Hygrometer; (**C**) total oral condition score; (**D**) swallowing function assessed by the RSST. Data are presented as group means with 95% confidence intervals.

As shown in Figure 2, all four predefined primary outcomes demonstrated consistent improvement during 12 weeks of NYT treatment. The mean symptom severity score decreased from 8.27 at baseline to 7.45 at 4 weeks, 6.00 at 8 weeks, and 3.64 at 12 weeks, with repeated-measures ANOVA indicating significant reductions at 8 weeks (*p* = 0.029) and 12 weeks (*p* = 0.006). Oral mucosal moisture increased progressively from 25.09 at baseline to 28.07 at week 12, exceeding the clinical threshold of 28.0, and reached statistical significance (*p* = 0.03). Oral condition scores improved markedly from 5.09 at baseline to 1.36 at 12 weeks (*p* = 0.006, Friedman test). RSST swallowing frequency rose from 2.18 at baseline to 4.55 at 12 weeks, surpassing the diagnostic cut-off of three swallows/30 s and showing a significant increase (*p* = 0.046). These improvements were observed consistently across multiple domains, including systemic symptom burden, oral moisture status, clinician-assessed oral condition, and swallowing function, indicating a broad effect of NYT on both general and oral health-related outcomes.

To further quantify the longitudinal changes observed in the primary outcomes, linear mixed-effects (LME) regression analysis was performed, and the detailed results are summarized in Table 3. The analysis demonstrated significant time-dependent reductions in symptom severity (Coef. = −0.384, 95% CI −0.493 to −0.275, *p* < 0.001) and oral condition scores (Coef. = −0.314, 95% CI −0.432 to −0.195, *p* < 0.001). In contrast, oral mucosal moisture (Coef. = +0.220, 95% CI +0.093 to +0.347, *p* < 0.001) and RSST swallowing frequency (Coef. = +0.186, 95% CI +0.084 to +0.289, *p* < 0.001) increased significantly over time. Random-effect parameters, including the standard deviations of the intercept and slope, indicated inter-individual variability in baseline status and rates of change; however, the overall pattern consistently supported clinically meaningful and temporally consistent improvements in primary outcomes during NYT treatment.

### 3.3. Changes in Taste Function and Oral Function Questionnaire

**Figure 3:**
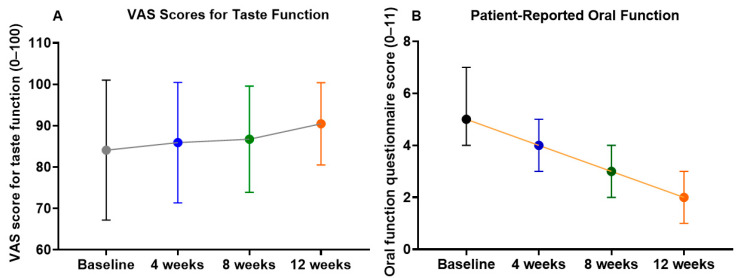
Changes in patient-reported taste and oral function during 12 weeks of NYT treatment. (**A**) taste function assessed using a VAS; (**B**) patient-reported oral function assessed using the 11-item questionnaire. Values represent group means with 95% confidence intervals.

As shown in Figure 3, the VAS score for taste function exhibited only a modest upward trend during the 12-week treatment. The mean score increased from 84.1 ± 25.2 at baseline to 85.9 ± 21.7 at 4 weeks, 86.7 ± 19.1 at 8 weeks, and 90.5 ± 14.8 at 12 weeks. These changes did not reach statistical significance (Friedman test, *p* = 0.21). A substantial proportion of participants reported high baseline taste function scores (≥80/100), suggesting a ceiling effect that may have limited the ability to detect further improvements at the group level. Notably, one participant (male patient No. 11) with a markedly low baseline score (16/100) demonstrated a progressive and clinically meaningful increase to 53/100 at week 12. Linear mixed-effects regression analysis (Table 3) confirmed this finding, showing a non-significant overall trend (Coef. = +0.498, 95% CI −0.071 to +1.066, *p* = 0.086).

In contrast, the oral function questionnaire scores (0–11) demonstrated significant improvement during NYT treatment. The mean score declined from 5.1 ± 1.3 at baseline to 4.0 ± 1.2 at 4 weeks, 3.2 ± 1.2 at 8 weeks, and 2.0 ± 0.6 at 12 weeks, with the Friedman test revealing a significant overall difference across time points (*p* < 0.001). Post hoc pairwise comparisons showed that scores at 8 and 12 weeks were significantly lower than baseline (*p* < 0.01 and *p* < 0.001, respectively), indicating progressive improvement in subjective oral function symptoms. LME regression (Table 3) supported this result, with a significant time-dependent reduction (Coef. = −0.252, 95% CI −0.317 to −0.187, *p* < 0.001).

Together, these results suggest that while the effect of NYT on taste function was limited at the group level, likely due to a ceiling effect, patient-reported oral function showed robust and progressive improvement during the intervention period.

### 3.4. Safety Outcomes

No serious adverse events were observed during the study period. In addition, no significant differences were observed in hematological and biochemical parameters after 12 weeks of NYT administration (Table 4). Most values, including WBC, hemoglobin, hematocrit, platelet counts, and renal function markers (CRE, BUN), remained stable throughout the study period.

Inflammatory and hepatic markers showed minor fluctuations: CRP levels increased slightly but remained within the normal range. Liver enzymes (AST, ALT) exhibited an upward trend; however, their values stayed within the normal reference range, and the changes did not reach statistical significance.

Notably, the lipid profile demonstrated a favorable tendency. LDL-C levels decreased, while HDL-C levels increased, with HDL-C approaching statistical significance (*p* = 0.057). Triglycerides also showed a slight decrease. Together, these findings suggest that the NYT administration was not associated with adverse hematological or biochemical effects and may contribute to modest improvements in lipid metabolism.

### 3.5. Immunological Outcomes

**Figure 4:**
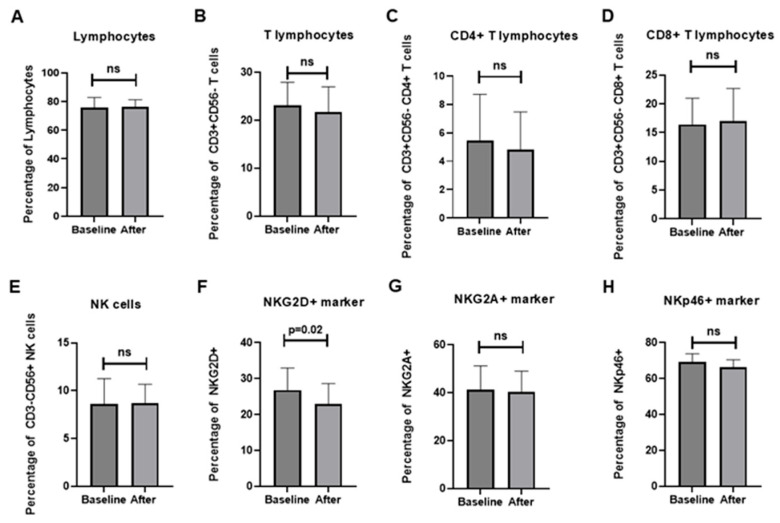
Effects of NYT treatment on lymphocyte and NK/T-cell subsets: (**A**) total lymphocytes; (**B**) CD3^+^CD56^−^ T cells; (**C**) CD4^+^ T cells; (**D**) CD8^+^ T cells; (**E**) CD3^−^CD56^+^ NK cells; (**F**) NKG2D^+^ cells; (**G**) NKG2A^+^ cells; (**H**) NKp46^+^ cells. Data are presented as mean ± SD before and after 12 weeks of treatment. ns, not significant; *p* < 0.05 was considered statistically significant.

**Figure 5:**
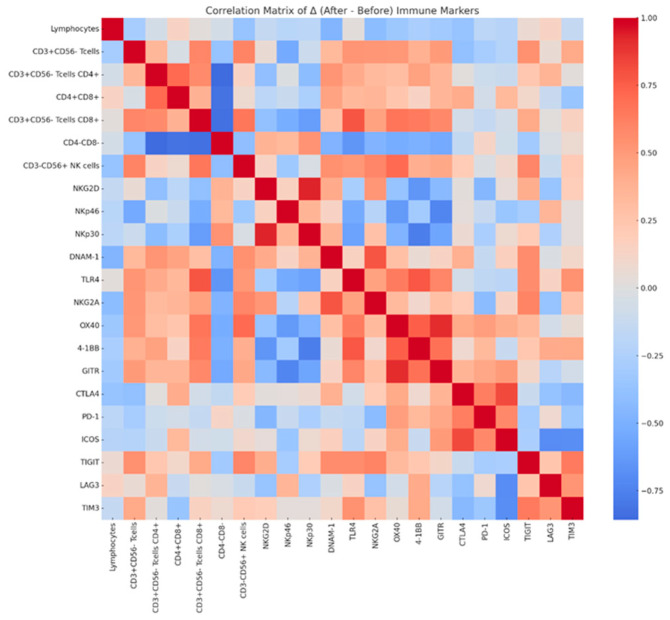
Correlation matrix of changes (Δ) in immune cell subsets and receptor expression before and after NYT administration. The heatmap shows Pearson correlation coefficients between changes in immune cell populations and regulatory markers, with red indicating positive correlations, blue indicating negative correlations, and lighter colors indicating weaker correlations.

The overall proportion of lymphocytes remained stable during the intervention (75.8% ± 7.1 at baseline vs. 76.4% ± 4.9 after treatment, *p* = 0.807). CD3^+^CD56^−^ T cells decreased modestly (23.1% ± 4.9 vs. 21.7% ± 5.3, *p* = 0.398), with a slight downward trend in the CD4^+^ subset (5.44% ± 3.3 vs. 4.81% ± 2.7, *p* = 0.366), whereas CD8^+^ T cells remained unchanged (16.4% ± 4.6 vs. 17.0% ± 5.7, *p* = 0.699). CD3^−^CD56^+^ NK cells also showed no significant differences (Figure 4). Among activating receptors, NKG2D expression decreased significantly from 26.8% ± 6.2 to 22.9% ± 5.8 (Δ = −3.94, 95% CI: −7.91 to −0.03, *p* = 0.026). NKp46^+^ and NKp30^+^ cells demonstrated downward but non-significant trends, while inhibitory receptors, including NKG2A, CTLA-4, PD-1, and TIGIT, remained stable. GITR showed a non-significant tendency to increase (Δ = +8.11 ± 12.83, *p* = 0.077). Detailed numerical values for all immune markers, including additional co-stimulatory and inhibitory receptors (e.g., TLR4, ICOS, LAG-3, TIM-3), are presented in Supplementary Table S4. Correlation matrix analysis of Δ values revealed weak-to-moderate positive associations among most markers, suggesting preservation of overall immune network balance following NYT treatment. Notably, reductions in NKG2D correlated negatively with changes in CD3^+^CD56^−^CD4^+^ T cells, whereas other co-stimulatory and inhibitory receptors showed minimal inter-relationships (Figure 5).

## 4. Discussion

Our findings suggest that NYT significantly alleviates physical symptoms such as fatigue, anorexia, irritability, and drowsiness, as previously reported [27]. Among its components, Panax ginseng is a well-known herbal treatment for chronic fatigue syndrome, with established efficacy and safety [28]. Ogawa-Ochiai et al. also demonstrated that Panax ginseng reduces fatigue in patients with frailty and significantly enhances NK cell activity [29,30].

*Atractylodes macrocephala*, another major NYT component, is traditionally used to address digestive and gastrointestinal issues. It promotes gastric emptying by stimulating stomach and intestinal activity, while its macrocephalic lactone I content enhances salivary amylase activity, intestinal absorption, and overall gastrointestinal regulation [31]. These properties may explain the capacity of NYT in reducing appetite loss. Furthermore, Schisandra chinensis has demonstrated its ability to improve endurance and energy metabolism in exercised rats. This effect is linked to the upregulation of peroxisome proliferator-activated receptor-gamma coactivator-1α, a key regulator of skeletal muscle energy metabolism [32]. Additionally, polysaccharides and α-cubebenoate derived from Schisandra fruit exhibit anti-fatigue effects through mechanisms such anti-inflammatory activity [33].

This study also revealed a substantial reduction in oral symptom severity, including improved oral mucosal moisture following NYT treatment. Enhanced mucosal moisture reduces the risk of xerostomia (dry mouth), a condition associated with complications such as bad breath, poor oral hygiene, dental decay, and difficulties in speaking and swallowing. Consistent with prior research, NYT has shown potential in alleviating dry mouth symptoms [34]. It can be observed that after treatment with NYT, there have been physical changes in the oral symptoms of elderly individuals. Recent studies have demonstrated that chewing, swallowing, digestion, and nutrient absorption are interconnected processes [35]. Oropharyngeal dysphagia, characterized by difficulties in swallowing and food residue post-swallowing, increases with age and poses serious health issues, including malnutrition [36]. In this study, NYT not only mitigated dry mouth but also improved speech clarity and swallowing function. These results suggest that NYT may contribute to the improvement of oral health and overall quality of life in elderly individuals. Moreover, considering NYT as a supplementary intervention to improve nutritional intake merits further investigation.

The cost-effective, innocuous, and simple nature of the RSST makes it an optimal screening tool for detecting dysphagia in various patient populations. In this study, swallowing frequency on the RSST significantly increased following NYT treatment, suggesting a beneficial effect for patients with swallowing dysfunction. This improvement holds particular significance for elderly or stroke patients, as aging is the strongest risk factor for stroke [37,38]. Dysphagia affects more than 50% of stroke survivors [39]. These results indicate that NYT could enhance swallowing ability in elderly patients and individuals recovering from a stroke. Given the interrelationship between age, stroke, and swallowing dysfunction, NYT shows potential for improving functional symptoms and nutritional status in this population. Additionally, swallowing is critical to maintaining the body’s nutritional functions. The observed improvement in swallowing frequency following NYT treatment offers hope that this traditional herbal medicine could become an effective intervention for alleviating functional symptoms and enhancing nutritional intake in elderly individuals, particularly those with stroke-related dysphagia.

In addition to objective findings, subjective symptom burden was also evaluated using the VAS and the 11-item questionnaire. Both instruments showed consistent trends toward improvement in domains such as fatigue, loss of appetite, and irritability, supporting the clinical relevance of NYT in reducing patient-perceived discomfort. These subjective assessments complement the objective measures, highlighting the holistic benefits of NYT for elderly patients with multiple complaints.

In this study, we observed a modest reduction in total T lymphocytes, primarily driven by a decline in the CD4^+^ T-cell population, while CD8^+^ T cells remained stable. NKG2D expression also decreased following NYT administration. Because NKG2D is expressed across multiple lymphocyte subsets—including NK cells, CD8^+^ T cells, CD4^+^CD8^+^ double-positive cells, and CD4^+^ T cells—it is important to consider which population underlies this change. Our correlation analysis did not reveal a strong or definitive association between NKG2D expression and any specific T-cell subset. Nevertheless, previous studies have highlighted that aberrant NKG2D expression on CD4^+^ T cells is a hallmark of immune aging, whereas CD4^+^CD8^+^ double-positive cells are rare in peripheral blood and unlikely to account for the observed reduction [40,41]. Thus, while our findings suggest a parallel decline in CD4^+^ T cells and NKG2D, further investigation is required to clarify whether these phenomena are mechanistically linked.

The immune system plays a central role in defending against pathogens and malignancies. CD8^+^ T cells and NK cells are well-established mediators of tumor rejection through interferon-γ production and cytotoxicity [42]. CD4^+^ T cells are equally essential for orchestrating immune responses. Prior studies have reported the presence of NKG2D^+^CD4^+^ cells in patients with cancer, autoimmune disease, or chronic infection [40,43,44,45]. In the elderly, naïve T-cell compartments decline while memory populations increase, with aberrant CD4^+^NKG2D^+^ cells emerging as a feature of immunosenescence [40,46,47]. Unlike NKG2D^+^ NK or CD8^+^ cells, CD4^+^NKG2D^+^ cells exert immunosuppressive effects by inhibiting T-cell activity through Fas ligand pathways [48]. Accordingly, a reduction in such cells could theoretically mitigate immune aging and restore immune competence. Prior animal studies have shown that NYT decreases CD4^+^ T-cell levels in tumor models [49], and miR-16 has been implicated in regulating CD4^+^NKG2D^+^ activity by directly targeting NKG2D [50]. While our data cannot conclusively attribute NKG2D reduction to CD4^+^ subsets, the possibility that NYT modulates this pathway remains of interest.

Taken together, these results indicate that NYT produced only limited shifts in immune markers, maintaining global immune balance while modestly reducing CD4^+^ T cells and NKG2D expression. This dual effect may reflect a subtle attenuation of senescent or suppressive CD4^+^NKG2D^+^ cells, though confirmatory mechanistic studies are needed. Importantly, most other immune markers—including activating receptors such as NKp46 and NKp30, as well as inhibitory checkpoints such as PD-1, TIM-3, and TIGIT—remained stable over the 12-week treatment period, indicating that NYT did not induce broad immunosuppression but rather exerted a selective regulatory effect on specific immune subsets associated with aging. Such selective modulation may represent a fine-tuning mechanism that helps maintain immunological balance. Although a modest reduction in total T lymphocytes—mainly CD4^+^ T cells—was observed, this change remained within age-appropriate physiological ranges and did not coincide with any clinical signs of immunosuppression. In frail older adults, such fluctuations often reflect immunoregulatory adjustments rather than immune deterioration, particularly when other key immune markers remained stable. Nonetheless, given the small sample size and exploratory design, this finding should be interpreted with caution. Larger studies with detailed immunophenotyping are required to determine whether this reduction represents a beneficial modulation of senescent CD4^+^NKG2D^+^ cells or simply natural biological variability.

No clinically relevant adverse changes were observed in hematological or biochemical parameters, supporting the overall safety of NYT in elderly patients. Moreover, favorable trends in lipid metabolism were noted, suggesting that NYT may also contribute to maintaining cardiovascular health, although these findings warrant further confirmation in larger studies.

The participants in this study presented with multiple comorbidities and extensive polypharmacy, both of which may have influenced baseline oral function, immune status, and responsiveness to NYT. Common comorbidities included hypertension, dyslipidemia, diabetes mellitus, osteoporosis, glaucoma, rheumatoid arthritis, spinal canal stenosis, coronary arteriosclerosis, post-stroke sequelae, pulmonary MAC disease, and various inflammatory or autoimmune conditions. Many patients were also receiving multiple concomitant medications such as antihypertensives, statins, antidiabetic agents, psychotropic drugs, proton-pump inhibitors, bisphosphonates, analgesics, magnesium preparations, and antirheumatic therapies. Several of these medications—particularly psychotropics, antihypertensives, and PPIs—are known to cause xerostomia, impair taste perception, alter swallowing function, or modulate immune activity [51,52,53]. Therefore, both comorbidity burden and polypharmacy may act as important confounders that contribute to variability in oral and immunological outcomes. As this pilot study was not powered to adjust for these factors, larger studies will be necessary to determine their independent and combined influence on treatment effects.

This study has several limitations. First, the sample size was very small (*n* = 11), which limits the statistical power and generalizability of the findings. Because of this limited cohort size and the imbalance in sex distribution (only two male participants), adjustments for covariates such as age and gender were not feasible, and all statistical comparisons should be interpreted as exploratory. Second, the questionnaires used to assess symptom severity and oral condition were developed by the clinical team and have not undergone formal psychometric validation; although similar oral frailty screening tools have shown validity in previous studies, the absence of formal validation for the instruments used here remains a limitation. Third, detailed oral prosthodontic information (e.g., presence of fixed partial dentures, removable partial dentures, or complete dentures) was not systematically recorded, which may affect the interpretation of oral functional outcomes. Fourth, immune assessments were limited to peripheral blood and examined only selected immune-cell markers; more extensive immunophenotyping—including analyses of naïve/memory subsets, senescent markers, and tissue-level immune responses—was not performed. Fifth, although a modest reduction in CD4^+^ T cells was observed, this pilot study was not designed to evaluate immunological safety endpoints, and the clinical significance of this finding remains uncertain. Finally, although an FDR correction was applied for immune-cell markers, the analyses remain exploratory due to multiple comparisons and small sample size. Larger, controlled studies with comprehensive clinical and immunological assessments are required to confirm and extend these preliminary observations.

## 5. Conclusions

This pilot study suggests that NYT administration may be associated with gradual improvements in oral and nutritional symptoms among elderly individuals with impaired oral function. Participants reported reduced fatigue and swallowing difficulties, indicating potential benefits of NYT for supporting oral health and nutritional maintenance in older adults. Additionally, exploratory immune analyses hinted at mild modulatory effects without global suppression, which could be relevant for future mechanistic investigations. Overall, these findings provide preliminary insight into the possible role of NYT in promoting oral health and general well-being among the elderly, highlighting the need for larger controlled studies to confirm its efficacy and clarify underlying mechanisms.

## Figures and Tables

**Table 1:** Baseline characteristics of study participants.

No.	Sex	Age	Height (cm)	Comorbidities	Concomitant Medication(s)
1	F	74	163.3	Obstructive arteriosclerosis	Tsumura Keishibukuryogan with Yokuinin 5 g (Tsumura & Co., Tokyo, Japan), Saffron 0.8 g
2	F	88	143	Hypertension, osteoporosis, allergic rhinitis, chronic pharyngolaryngitis, postoperative left femoral fracture	Tramadol/acetaminophen (Tramacet; Janssen Pharmaceuticals, Beerse, Belgium), Eldecalcitol (Edirol; Chugai Pharmaceutical, Tokyo, Japan), Minodronate, Diclofenac, Carbocisteine, Levocetirizine (Xyzal; UCB Pharma, Brussels, Belgium), Amlodipine/Irbesartan (Aimix; Daiichi Sankyo, Tokyo, Japan), Vonoprazan (Takecab; Takeda Pharmaceutical, Tokyo, Japan), Atorvastatin, Arozein, Etizolam (Depas; Mitsubishi Tanabe Pharma, Osaka, Japan), Azunol gargle solution
3	F	81	148	Hypertension, dyslipidemia, insomnia, spinal canal stenosis	Esomeprazole (Nexium; AstraZeneca, Cambridge, UK), Etizolam (Depas; Mitsubishi Tanabe Pharma, Osaka, Japan), Amlodipine, Atorvastatin, Camia, Magnesium oxide (Magmitt; Nichi-Iko Pharmaceutical, Toyama, Japan), Ketoprofen tape, Rionsal, Acetaminophen (Calonal; Astellas Pharma, Tokyo, Japan), Neurobion, Calfina, Mornas tape
4	F	84	159	Hypertension, dyslipidemia, insomnia	Bezafibrate, Amlodipine, Olmesartan, Suvorexant (Belsomra; MSD, Kenilworth, NJ, USA), Zolpidem (Myslee; Astellas Pharma, Tokyo, Japan), Flavitan, Cinal.
5	F	77	156	None	None
6	M	70	153	Heberden’s nodes	Keishakubokuto 9 g (Tsumura & Co., Tokyo, Japan), Tsumura Bushi powder 1.5 g (Tsumura & Co., Tokyo, Japan).
7	F	75	173	Allergic rhinitis, glaucoma, arrhythmia	Desloratadine (Desalex; Organon, Oss, Netherlands), Flecainide acetate (Sunrythm; Eisai, Tokyo, Japan) (as needed)
8	F	72	153.5	Post-right frontal subcortical hemorrhage, hypertension, reflux esophagitis, liver dysfunction, delusional disorder	Candesartan, Calnedin, Nifedipine, Lansoprazole, Magnesium oxide (Magmitt; Nichi-Iko Pharmaceutical, Toyama, Japan), Brotizolam (Lendormin; Eisai, Tokyo, Japan), Benzarin, Risperidone, Levomepromazine (Levotomin; Sumitomo Pharma, Osaka, Japan), Vapro, Purzenid
9	F	73	150	HBV carrier, rheumatoid arthritis, cranial meningioma, constipation	Methotrexate, Entecavir (Baraclude; Bristol Myers Squibb, New York, NY, USA), Magnesium oxide.
10	F	71	153	Type 2 diabetes mellitus, localized scleroderma, pulmonary MAC disease, dyslipidemia, past hepatitis B infection, coronary arteriosclerosis	Procirin, Tocopherol nicotinate, Rebamipide, Mosapride citrate tablets, Triazolam, Diazepam, Alprazolam, Bosentan (Tracleer; Actelion Pharmaceuticals, Allschwil, Switzerland), Flunitrazepam, Mitiglinide (Glufast; Sumitomo Pharma, Osaka, Japan), Eicosapentaenoic acid ethyl ester (Epadel S900; Mochida Pharmaceutical, Tokyo, Japan), Ezetimibe (Zetia; MSD, Kenilworth, NJ, USA), Atorvastatin, Linagliptin (Equa; Boehringer Ingelheim, Ingelheim am Rhein, Germany), Aspirin (Bayer; Leverkusen, Germany), Rabeprazole (Pariet; Eisai, Tokyo, Japan), Erythromycin, Carbocisteine
11	M	75	166	Diabetes mellitus, dyslipidemia, bulbar palsy, glaucoma, post-gastric polyp resection, prostatic hypertrophy, open nasal voice	Metformin (Metgluco; Sumitomo Pharma, Osaka, Japan), Atorvastatin (Lipitor; Pfizer, New York, NY, USA)

**Table 2:** Component ratios of Ninjinyoeito supplementation.

Component	Relative Ratio (*w*/*w*)
*Panax ginseng* C.A. Meyer	3.0
*Angelica acutiloba* Kitagawa	4.0
*Paeonia lactiflora* Pallas	2.0
*Rehmannia glutinosa* Liboschitz	4.0
*Atractylodes macrocephala* Koidzumi	4.0
*Wolfiporia cocos* Ryvarden et Gilbertson	4.0
*Cinnamomum cassia* Blume	2.5
*Astragalus mongholicus* Bunge	1.5
*Citrus unshiu* Marcowicz	2.0
*Polygala tenuifolia* Willdenow	2.0
*Schisandra chinensis* Baillon	1.0
*Glycyrrhiza uralensis* Fischer	1.0

Ratio indicates the relative weight proportion of each crude drug in the formulation. The component names of the product have been verified with the website http://www.theplantlist.org (accessed on 30 November 2025).

**Table 3:** Linear mixed-effects regression results for primary outcomes and secondary outcomes.

Outcome	Coef.	SD Intercept	SD Slope	*p*-Value	95% CI
Symptom severity	−0.384	2.35	0.144	<0.001	−0.493; −0.275
Oral condition	−0.314	2.10	0.179	<0.001	−0.432; −0.195
Oral moisture	+0.220	1.02	0.144	<0.001	+0.093; +0.347
RSST	+0.186	0.98	0.140	<0.001	+0.084; +0.289
VAS score for taste function (0–100)	+0.498	14.39	0.79	0.086	−0.071; +1.066
Oral function questionnaire (0–11)	−0.252	0.89	0.05	<0.001	−0.317; −0.187

**Table 4:** Hematological and biochemical parameters before and after NYT treatment.

	Baseline Mean ± SD	After Mean ± SD	Δ Mean	95% CI	*p*-Value
WBC (×10^3^/µL)	5.33 ± 1.03	5.39 ± 1.21	0.055	[−0.469, 0.580]	0.818
Hb (g/dL)	13.22 ± 1.55	13.21 ± 1.72	−0.009	[−0.357, 0.339]	0.955
Hct (%)	39.98 ± 4.36	40.25 ± 5.00	0.273	[−0.677, 1.223]	0.537
PLT (×10^3^/µL)	232.00 ± 48.40	235.18 ± 63.35	3.182	[−16.030, 22.393]	0.720
RDW (%)	13.39 ± 0.88	13.51 ± 0.95	0.118	[−0.142, 0.378]	0.335
Neutrophils (%)	58.68 ± 8.28	59.26 ± 8.34	0.583	[−2.649, 3.814]	0.696
Lymphocytes (%)	32.41 ± 8.80	32.46 ± 8.68	0.055	[−3.110, 3.220]	0.970
Monocytes (%)	6.04 ± 1.77	5.82 ± 1.26	−0.218	[−1.308, 0.872]	0.665
NLR	2.01 ± 0.85	2.01 ± 0.80	−0.002	[−0.356, 0.352]	0.989
LMR	5.84 ± 2.36	5.82 ± 2.14	−0.022	[−0.947, 0.904]	0.960
PLR	7.83 ± 3.63	7.87 ± 3.44	0.033	[−0.917, 0.982]	0.940
CRP	0.12 ± 0.14	0.17 ± 0.22	0.054	[−0.018, 0.126]	0.122
AST	26.82 ± 11.48	30.64 ± 16.79	3.818	[−0.949, 8.586]	0.105
ALT	22.36 ± 13.99	26.91 ± 19.16	4.545	[−0.965, 10.056]	0.096
γ-GTP	25.00 ± 26.02	28.45 ± 40.21	3.455	[−6.361, 13.270]	0.451
T-Bil	0.50 ± 0.15	0.50 ± 0.16	−0.000	[−0.052, 0.052]	1.000
CRE	0.72 ± 0.17	0.68 ± 0.14	−0.034	[−0.104, 0.037]	0.312
BUN	14.64 ± 4.88	14.64 ± 3.47	0.000	[−1.876, 1.876]	1.000
LDL-C	100.90 ± 28.71	92.80 ± 24.73	−8.100	[−18.659, 2.459]	0.117
HDL-C	54.70 ± 14.01	56.20 ± 14.05	1.500	[−0.055, 3.055]	0.057
TG	134.00 ± 51.51	130.50 ± 50.25	−3.500	[−49.255, 42.255]	0.866
SBP	121.64 ± 10.90	120.55 ± 9.16	−1.091	[−9.059, 6.878]	0.767
DBP	65.73 ± 10.85	66.91 ± 9.58	1.182	[−6.483, 8.846]	0.738

WBC, white blood cells; Hb, hemoglobin; Hct, hematocrit; PLT, platelets; RDW, red cell distribution width; NLR, neutrophil-to-lymphocyte ratio; LMR, lymphocyte-to-monocyte ratio; PLR, platelet-to-lymphocyte ratio; CRP, C-reactive protein; AST, aspartate aminotransferase; ALT, alanine aminotransferase; γ-GTP, gamma-glutamyl transpeptidase; T-Bil, total bilirubin; CRE, creatinine; BUN, blood urea nitrogen; LDL-C, low-density lipoprotein cholesterol; HDL-C, high-density lipoprotein cholesterol; TG, triglycerides; SBP, systolic blood pressure; DBP, diastolic blood pressure.

## Data Availability

The materials described in this study’s findings, including all relevant raw data, will be freely available to any scientist for non-commercial use upon request to the corresponding author, provided that patient confidentiality is not compromised.

## Supplementary Materials

The following supporting information can be downloaded at: https://www.mdpi.com/article/10.3390/medicina62010048/s1, Table S1: Severity of Symptoms Questionnaire (example for one patient); Table S2: Oral Condition Assessment Questionnaire (example for one patient); Table S3: 11-item Questionnaire for Oral Function (example for one patient); Table S4: Immune-related markers before and after 12 weeks of NYT administration.

## Abbreviations

The following abbreviations are used in this manuscript:

ALT	Alanine aminotransferase
AST	Aspartate aminotransferase
BUN	Blood urea nitrogen
Cl	Chloride
CRE	Creatinine
CRP	C-reactive protein
DBP	Diastolic blood pressure
FDR	False Discovery Rate
Hb	Hemoglobin
HDL-C	High-density lipoprotein cholesterol
Ht	Hematocrit
K	Potassium
LME	Linear mixed-effects (regression)
LDL-C	Low-density lipoprotein cholesterol
LDH	Lactate dehydrogenase
LMR	Lymphocyte-to-monocyte ratio
MCH	Mean corpuscular hemoglobin
MCHC	Mean corpuscular hemoglobin concentration
MCV	Mean corpuscular volume
Na	Sodium
NK	Natural killer (cells)
NLR	Neutrophil-to-lymphocyte ratio
NYT	Ninjin’yoeito
PBMCs	Peripheral blood mononuclear cells
PDW	Platelet distribution width
PLR	Platelet-to-lymphocyte ratio
PLT	Platelet count
RDW	Red cell distribution width
RSST	Repetitive saliva swallowing test
SBP	Systolic blood pressure
T-Bil	Total bilirubin
TG	Triglycerides
VAS	Visual analogue scale
WBC	White blood cell
γ-GTP	Gamma-glutamyl transpeptidase
