# Supplementary material for: Ninjin’yoeito for Impaired Oral Function in Older Adults: A Prospective, Open-Label Pilot Study
Source: Medicina (Kaunas). 2025 Dec 26;62(1):48. doi: 10.3390/medicina62010048 (PMC12843259; doi:10.3390/medicina62010048)
Supplement: Supplementary file 1 [file medicina-62-00048-s001.zip › Supplementary Table S1.pdf]

Supplementary Table 1. *Severity of Symptoms Questionnaire* (Example for one patient).

Scores: 3 = Severe, 2 = Moderate, 1 = Mild, 0 = None.

The total score (0–18) represents the sum of all six symptoms.

**Symptom**	**Baseline**	**Week 4**	**Week 8**	**Week 12**
Physical decline after illness	2	2	2	1
Fatigue / Lassitude	2	2	2	1
Loss of appetite	2	2	1	0
Somnolence (sleepiness)	0	0	0	0
Cold extremities	0	0	0	0
Anemia	0	0	0	0
**Total (0–18)**	**6**	**6**	**5**	**2**
