# Supplementary material for: Ninjin’yoeito for Impaired Oral Function in Older Adults: A Prospective, Open-Label Pilot Study
Source: Medicina (Kaunas). 2025 Dec 26;62(1):48. doi: 10.3390/medicina62010048 (PMC12843259; doi:10.3390/medicina62010048)
Supplement: Supplementary file 1 [file medicina-62-00048-s001.zip › Supplementary Table S2.pdf]

Supplementary Table 2. *Oral Condition Assessment Questionnaire* (Example for one patient).

Scores: 3 = Severe, 2 = Moderate, 1 = Mild, 0 = None.

The total score (0–15) represents the sum of all five items.

**Item (Oral condition)**	**Baseline**	**Week 4**	**Week 8**	**Week 12**
Oral deposits/discoloration (teeth, dentures, tongue)	0	0	0	0
Halitosis (bad breath)	0	0	0	0
Lack of facial expression around mouth	1	1	1	1
Speech problems (unclear articulation, dysarthria, reduced conversation)	1	1	1	0
Residual food after swallowing	1	2	1	1
**Total (0–15)**	**3**	**4**	**3**	**2**
