# Supplementary material for: Ninjin’yoeito for Impaired Oral Function in Older Adults: A Prospective, Open-Label Pilot Study
Source: Medicina (Kaunas). 2025 Dec 26;62(1):48. doi: 10.3390/medicina62010048 (PMC12843259; doi:10.3390/medicina62010048)
Supplement: Supplementary file 1 [file medicina-62-00048-s001.zip › Supplementary Table S3.pdf]

Supplementary Table 3. *11-item Questionnaire for Oral Function* (Example for one patient). Scoring: "Yes (◯)" = impaired condition (except Q11, where "Yes" = good condition). The total score represents the number of impaired conditions (0–10).

**Item**	**Baseline**	**Week 4**	**Week 8**	**Week 12**
1. Difficulty eating hard foods	◯	◯		
2. Choking on tea or soup			◯	
3. Mouth tends to dry easily	◯	◯		
4. Difficulty swallowing medication		◯		
5. Tongue gets caught while speaking				
6. Concern about bad breath	◯			
7. Prolonged mealtime				
8. Difficulty recognizing subtle flavors				
9. Food spillage while eating	◯	◯	◯	
10. Food residue remains in the mouth after meals	◯	◯	◯	◯
11. Ability to bite firmly with natural or prosthetic molars (good if ◯)		◯		
**Total impaired (0–10)**	**6**	**5**	**4**	**2**
