# Supplementary material for: Ninjin’yoeito for Impaired Oral Function in Older Adults: A Prospective, Open-Label Pilot Study
Source: Medicina (Kaunas). 2025 Dec 26;62(1):48. doi: 10.3390/medicina62010048 (PMC12843259; doi:10.3390/medicina62010048)
Supplement: Supplementary file 1 [file medicina-62-00048-s001.zip › Supplementary Table S4.pdf]

Supplementary Table 4. Immune-related markers before and after 12 weeks of NYT administration

**Group**	**Marker**	**Baseline Mean ± SD**	**After Mean ± SD**	**Δ Mean**	**Raw p**	**FDR (subgroup)**
**T-cell subsets**	Lymphocytes	75.83±7.06	76.42±4.86	0.590	0.807	**0.807**
	$CD3^{+}CD56^{-}$ T cells	23.06±4.88	21.67±5.33	-1.393	0.398	**0.786**
	$CD4^{+}$	5.44±3.28	4.81±2.67	-0.630	0.366	**0.786**
	$CD4^{+}CD8^{+}$	3.70±4.77	1.86±1.03	-1.833	0.195	**0.786**
	$CD8^{+}$	16.42±4.56	17.01±5.66	0.582	0.700	**0.807**
	$CD4^{-}CD8^{-}$	74.45±8.06	76.33±6.28	1.883	0.524	**0.786**
**NK cells**	$CD3^{-}CD56^{+}$ NK	8.64±2.62	8.72±1.95	0.078	0.911	**0.911**
**Activating NK receptors**	**NKG2D**	26.82±6.15	22.88±5.75	-3.940	**0.026**	**0.104**
	NKp46	69.42±4.44	66.48±4.00	-2.940	0.130	**0.173**
	NKp30	63.42±6.05	58.58±6.22	-4.840	0.092	**0.173**
	DNAM-1	11.16±5.96	10.46±5.10	-0.699	0.701	**0.701**
**TLR (innate)**	TLR4	61.25±5.87	59.17±5.09	-2.080	0.131	**0.131**
**Inhibitory checkpoints**	NKG2A	41.16±9.99	40.28±8.73	-0.880	0.803	**0.913**
	CTLA-4	21.44±6.14	19.07±5.48	-2.370	0.227	**0.913**
	PD-1	66.23±4.95	65.47±5.94	-0.760	0.588	**0.913**
	TIGIT	11.10±4.10	11.62±4.31	0.525	0.649	**0.913**
	LAG-3	58.07±6.84	57.89±4.11	-0.180	0.913	**0.913**
	TIM-3	26.12±4.99	29.01±10.57	2.890	0.338	**0.913**
**Co-stimulatory receptors**	OX40	18.81±5.76	21.46±4.82	2.650	0.357	**0.476**
	4-1BB	65.23±6.94	67.37±4.25	2.140	0.271	**0.476**
	GITR	51.16±9.51	59.27±7.59	8.110	0.077	**0.308**
	ICOS	57.91±8.63	55.19±11.94	-2.720	0.495	**0.495**

FDR values represent subgroup-specific Benjamini–Hochberg corrections. Markers were grouped a priori based on established immunological pathways (T-cell subsets, NK-cell activation receptors, inhibitory checkpoints, co-stimulatory receptors, and TLR4). This approach prevents over-correction

Group	Marker	Baseline Mean ± SD	After Mean ± SD	Δ Mean	Raw p	FDR (subgroup)

across unrelated immune parameters and is recommended in hypothesis-driven exploratory studies with small sample sizes.
